# Appropriateness of lung ultrasound for the diagnosis of COVID‐19 pneumonia

**DOI:** 10.1002/hsr2.302

**Published:** 2021-05-24

**Authors:** Zouheir Ibrahim Bitar, Mohammed Shamsah, Ossama Sajeh Maadarani, Omar Mohammed Bamasood, Huda Al‐foudari

**Affiliations:** ^1^ Critical Care Unit, Ahmadi Hospital, Kuwait Oil Company Fahaheel Kuwait; ^2^ Adan Hospital, Intensive Care Unit Fahaheel Kuwait

**Keywords:** COVID‐19, lung ultrasound, pneumonia, RT‐PCR

## Abstract

**Background:**

Chest radiography (CXR) and computerized tomography (CT) are the standard methods for lung imaging in diagnosing COVID‐19 pneumonia in the intensive care unit (ICU), despite their limitations. This study aimed to assess the performance of bedside lung ultrasound examination by a critical care physician for the diagnosis of COVID‐19 pneumonia during acute admission to the ICU.

**Method:**

This was an observational, prospective, single‐center study conducted in the intensive care unit of Adan General Hospital from April 10, 2020, to May 26, 2020. The study included adults with suspicion of COVID‐19 Infection who were transferred to the ICU. Patients were admitted to the ICU directly from the ED after reverse transcriptase‐polymerase chain reaction (RT‐PCR) swabs were sent to the central virology laboratory in Kuwait, and the results were released 16 to 24 hours after the time of admission. A certified intensivist in critical care ultrasound performed the lung ultrasound within 12 hours of the patient's admission to the ICU.

The treating physician confirmed the diagnosis of COVID‐19 pneumonia based on a set of clinical features, inflammatory markers, biochemical profile studies, RT‐PCR test results, and CXR.

**Results:**

Of 77 patients with suspected COVID‐19 pneumonia, 65 (84.4%) were confirmed. The median age of the patients was 48 (31‐68) years, and 51 (71%) were men.

In the group of patients with confirmed COVID‐19 pneumonia, LUS revealed four signs suggestive of COVID‐19 pneumonia in 63 patients (96.9%) (sensitivity 96.9%, CI 85%‐99.5%). Two patients presented with unilateral lobar pneumonia without other ultrasonic signs of COVID‐19 pneumonia but with positive RT‐PCR results. Among patients in the group without COVID‐19 pneumonia who had negative RT‐PCR results, 11 (91.7%) were LUS negative for COVID‐19 pneumonia (specificity 91.7%, 95% CI 58.72%‐99.77%).

**Conclusions:**

During the COVID‐19 outbreak, LUS allows the identification of early signs of interstitial pneumonia. LUS patterns that show a combination of the four major signs offer high sensitivity and specificity compared to nasopharyngeal RT‐PCR.

## Graphical abstract

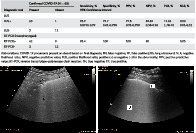

## 1 INTRODUCTION

Severe acute respiratory syndrome coronavirus 2 (SARS‐CoV‐2) infects cells in the lower respiratory tract,^1^ causing pneumonia as the primary complication of the disease. Chest radiography (CXR) and computerized tomography (CT) are the standard methods for lung imaging in diagnosing COVID‐19 pneumonia in the intensive care unit. The radiological picture of COVID‐19 pneumonia typically shows interstitial diffuse bilateral pneumonia with lesions exhibiting an asymmetric and patchy distribution in the lung periphery, a suitable site for ultrasound investigation.^2^

During the pandemic era of SARS‐CoV‐2, lung ultrasound has the advantage of being noninvasive and can be performed quickly at the bedside. Lung ultrasound can help diagnose cases with suspicion of COVID‐19 pneumonia in patients who have respiratory symptoms that necessitate urgent and early admission to the critical care area.

Looking for early sonographic signs of pneumonia upon admission can be helpful in the early diagnosis of COVID‐19 pneumonia. Herein, we tested the sensitivity and specificity of these signs in correlation with the standard test for SARS‐CoV‐2 disease with respiratory involvement.

## 2 METHODS

### 2.1 Study population

This was an observational, prospective, single‐center study conducted in the intensive care unit of Adan General Hospital from April 10, 2020, to May 10, 2020. The Ethical Committee of the Ministry of Health in Kuwait approved the study protocol, and informed consent was obtained from all patients or their next of kin.

**FIGURE 1:**
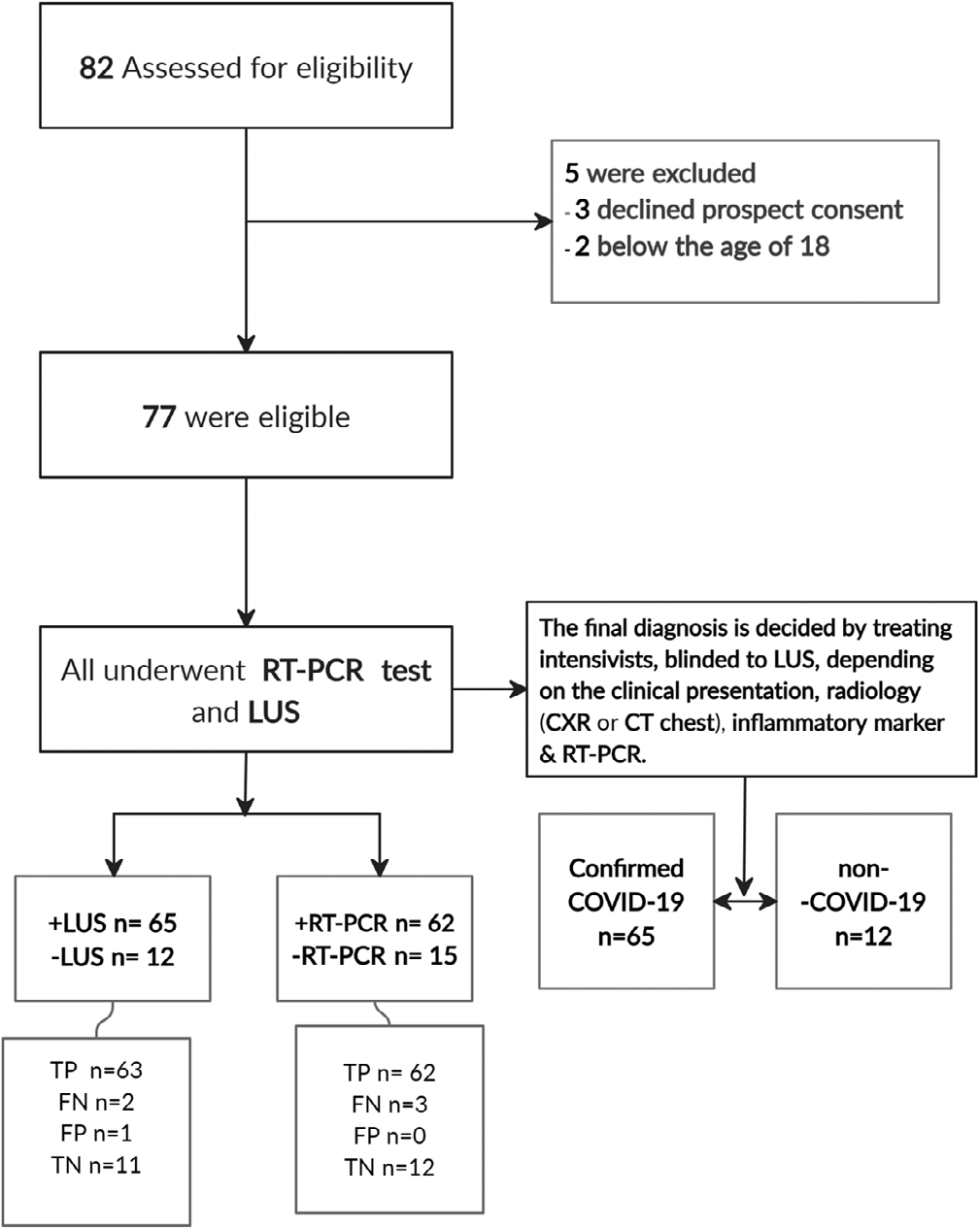
Flow diagram. LUS, lung ultrasound; RT‐PCR, reverse transcriptase–polymerase chain reaction; positive (+) or negative (−) for abnormality; CT, computerized tomography; CXR, chest‐X‐ray; TP, true positive; TN, true negative; TN, true negative; FN, false negative

Consecutive patients were included if they were >18 years of age with suspicion of COVID‐19 infection and had been transferred to the ICU with fever or suspected respiratory infection plus one of the following: respiratory rate > 30 breaths/min, severe respiratory distress, and SpO_2_ <93% on room air.^3^ Patients were admitted to the ICU directly from the ED after nasopharyngeal samples were collected for reverse transcriptase‐polymerase chain reaction (RT‐PCR) and sent to the central virology laboratory in Kuwait, and the results were released 16 to 24 hours after swab performance. Clinical data were entered on a separate standardized data collection form at the time of patient enrollment by the treating critical care physician. Clinical data included the patient's age and sex, presenting symptoms, medical history, oxygen saturation from pulse oximetry, and chest radiograph. A level 4 operator (entrusted to act unsupervised) in critical care ultrasound who was blinded to the RT‐PCR results,^4^ if available at the time of examination, performed the lung ultrasound within 12 hours of the patient's admission to the ICU (Figure 1).

All included patients were prospectively evaluated until discharge. The final diagnosis was made by the physician in charge based on RT‐PCR, radiological reports (chest CT and CXR), clinical progress, inflammatory markers, and microbiology studies. We compared the ultrasound and RT‐PCR results with the final diagnosis made by physicians in charge.

If the initial upper respiratory sample result was negative and the suspicion for disease remained high, repeat testing generally occurred 24 hours twice after the initial testing, or a lower respiratory tract sample was collected (eg, sputum, bronchoalveolar lavage fluid, tracheal aspirate) if accessible. We also compared the results of chest CT and LUS with the final diagnosis made by the treating physician who was blinded to the results of the LUS.

### 2.2 Protocol

We performed lung ultrasonography for all patients admitted to the ICU with suspicion of COVID‐19 infection using a 12‐zone method.^5^, ^6^ There were six zones in each hemithorax: two anterior, two axillary, and two posterior. The anterior chest wall was defined as extending from the parasternal line to the anterior axillary line. This zone was divided into upper and lower regions in the third intercostal space. The lateral area from the anterior to the posterior axillary line was divided into upper and lower halves. The posterior zone was identified from the posterior axillary line to the paravertebral line. The ultrasound images were saved to a hard drive and reviewed by a senior intensivist trained in critical care ultrasound. Ultrasound was performed using a portable ultrasound machine (GE Vivid S6N, N‐3191 Horten, Norway) equipped with a 3.5‐MHz broadband curvilinear transducer. The probe was placed in an oblique position on the intercostal space, and the pleural line was centered in the middle of the image by adjusting the depth settings. The oblique position of the probe on the intercostal space allows visualization of a larger portion of the pleural line without interruption from rib shadows.

### 2.3 Measurements

**FIGURE 2:**
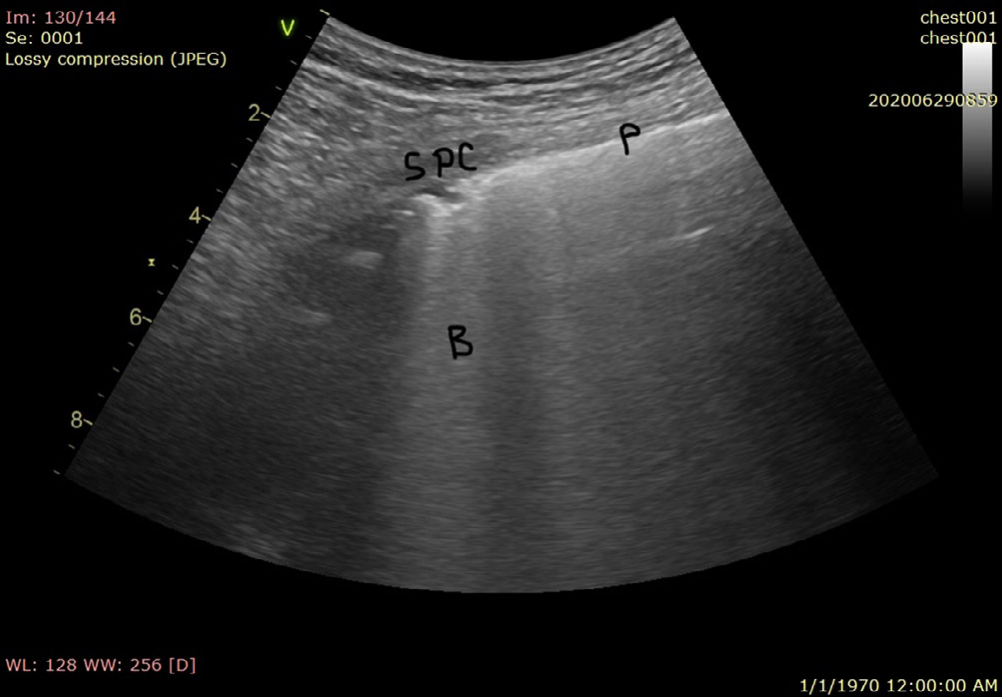
Multiple B lines arising from irregular pleura (P, pleura; B, B lines; SPC, subpleural consolidation)

**FIGURE 3:**
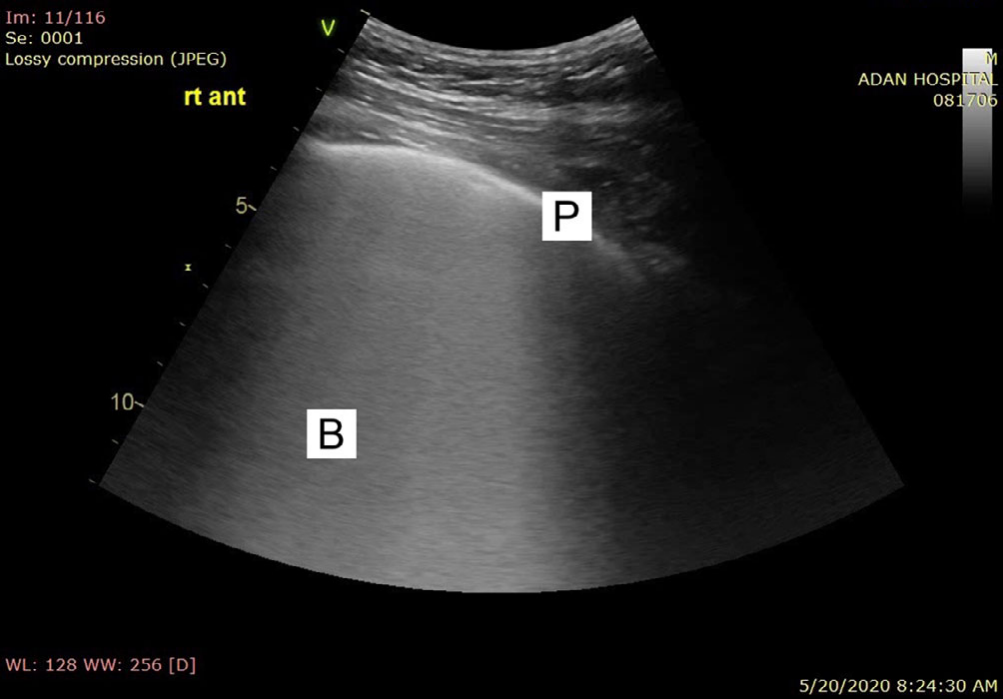
(P) Pleural line with patchy shining white lung areas

Pleural sliding and A‐lines (repetitive lines parallel to the pleural line) on ultrasound are seen in normal healthy lungs.^7^ Interstitial syndrome is indicated by the presence of multiple B lines (more than three lines in one region). The four signs of COVID‐19 pneumonia on lung ultrasound evaluation are as follows^8^ (Figures 2 and 3):

1. Bilateral B‐lines in separate forms and bilateral patchy shining white lung areas in multiform clusters, where all these signs are represented and sharply alternated to “spared areas”. The B lines maintain their brightness until the end of the screen. They arise either directly from limited sliding pleura or a small subpleural consolidation.
2. Bilateral diffuse irregularities of the pleural line.
3. Absence of significant pleural effusion.
4. Presence of multiple subpleural consolidations of various sizes (videos S1 and S2).

### 2.4 Data analysis

Statistical analyses were performed using SPSS 19. Sensitivity, specificity, and the positive and negative likelihood ratios of lung ultrasound and RT‐PCR for the diagnosis of COVID‐19 pneumonia were calculated. The McNamara test was used for dichotomous variables when appropriate *P* < .05 indicated significant differences. Our assumption of a result with 95% specificity and an approximate 1% prevalence and .05 confidence interval yielded an approximate optimal sample size of 90.^9^

## 3 RESULTS

Of 77 consecutive patients with suspected COVID‐19 pneumonia, 65 (84.4%) were confirmed (Figure 1). The median age of the patients was 48 (31‐68) years, and 51 (71%) were men. The clinical characteristics of the patients in relation to confirmed COVID‐19 pneumonia are shown in Table 1.

**TABLE 1:** Clinical characteristics of the patients in relation to ultrasound chest profiles

	Confirmed COVID‐19 65 cases (84.4%)	Non COVID‐19 12 cases (15.5%)	Total 77 cases (%)	*p* Value
Median age (IQR) ‐ years	48 (68‐31)	68 (25‐80)		
Male sex	51 (78%)	4 (33%)	55 (71%)	
Medical history				
IHD	3 (4.6%)	9 (75%)	12 (15.5%)	<0.0001
Hypertension	13 (20%)	10 (83%)	23 (30%)	0.99
Diabetes mellitus	15 (23%)	12 (100%)	27 (35%)	<0.0001
Asthma	1 (1%)	1 (8%)	2 (2%)	0.02
COPD	1 (1%)	1 (8%)	2 (2%)	0.164
Chronic renal impairment	4 (6%)	7 (58%)	11 (14%)	0.45
Cancer	0	1(8%)	1 (1%)	0.091
Status on admission to ICU				
Duration of symptoms (median in days)	5 (2–10)	2 (3–4)		
Hypoxemia	All	all	77 (100%)	0.02
HFNC	46 (71%)	4 (33%)	50 (64%)	0.037
IV	14 (21%)	6 (50%)	20 (26%)	<0.0001
Facemask	5 (7%)	2 (16.6%)	7 (9%)	<0.0001

Abbreviations: COPD, chronic obstructive pulmonary disease; HFNC, high‐flow nasal cannula; IHD, ischemic heart disease; IV, invasive ventilation.

In the group of patients with confirmed COVID‐19 pneumonia (Table 2), LUS revealed four signs suggestive of COVID‐19 pneumonia in 63 patients (96.9%; sensitivity 96.9%, CI 85%‐99.5%). Two patients presented with unilateral lobar pneumonia without other ultrasonic signs of COVID‐19 pneumonia but with positive nasopharyngeal samples for RT‐PCR. Of the patients in the group without COVID‐19 pneumonia, 11 (91.7%) were negative for LUS (specificity 91.7%, 95% CI 58.72%‐99.77%). One case declared positive by LUS with negative RT‐PCR was a case of non‐Hodgkin lymphoma postradiation pulmonary fibrosis and presented with hypertensive pulmonary edema. The other cases with negative LUS and negative RT‐PCR comprised three cases on chronic dialysis with pulmonary edema, one case of a 25‐year‐old woman with miliary tuberculosis and severe myocarditis, three cases of NSTEMI and pulmonary edema, one case of a 26‐year‐old man with uncontrolled hypertension, renal failure and possible vasculitis, one case of diabetic ketoacidosis and right basal pneumonia, and one case of hypertensive pulmonary edema with UTI.

**TABLE 2:** Lung ultrasound and RT‐PCR profiles of patients with respiratory distress and suspicion of COVID‐19 in ICU (N = 77)

	Confirmed COVID‐19 (N = 65)		95% Confidence interval					
Diagnostic tool	Present	Absent	Sensitivity, %	Specificity, %	PPV, %	NPV, %	PLR, %	NLR, %
LUS								
LUS+	63	1	96.9 0.89 to 0.99	91.7 0.61‐0.998	97.8 0.91‐0.998	84.62 0.58‐0.96	11.63 1.78‐76	0.03 0.01‐0.13
LUS‐	2	11						
RT‐PCR (Nasopharyngeal)								
RT‐PCR+	62	0	95.4	100	100	80		0.05
RT‐PCR‐	3	12						

Abbreviations: COVID‐19 pneumonia present or absent based on final diagnosis; FN, false negative; FP, false positive;LUS, lung ultrasound; NLR, negative likelihood ratio.; NPV, negative predictive value; PLR, positive likelihood ratio; positive (+) or negative (−) for the abnormality; PPV, positive predictive value; RT‐PCR, reverse transcriptase–polymerase chain reaction; TN, true negative; TP, true positive.

Three cases were considered COVID‐19 pneumonia by the treating team, despite three samples by nasopharyngeal swab 24 hours apart that returned negative RT‐PCR results, because their clinical pictures, lab findings, and CT chest findings were consistent with COVID‐19 pneumonia, and bronchoalveolar lavage fluid was positive by RT‐PCR. Pleural effusion was not observed in any patient with confirmed COVID‐19 pneumonia.

## 4 DISCUSSION

Chest imaging in COVID‐19 disease is important for early diagnosis and sometimes for prognosis. The ideal imaging test for this epidemic disease would be quick, deliverable at the bedside, reliable, reproducible, and have both high sensitivity and specificity. The main diagnostic modalities used in confirming COVID‐19 pneumonia in patients with positive RT‐PCR are CXR and CT, and a few centers utilize LUS. Small studies have evaluated the sensitivity and specificity of CT compared to nasopharyngeal RT‐PCR, and CT currently shows the highest sensitivity of any test for COVID‐19.^10^ Thoracic CT imaging has been proposed as a primary screening tool for COVID‐19 detection as it performs better than PCR.^10^ The radiological lung abnormalities found on CT may antedate the physical symptoms of COVID‐19; however, CT is a finite resource, exposes additional healthcare personnel to infected patients, and may not be available in some healthcare settings. Furthermore, decontamination protocols are not currently well defined and are time‐consuming. The practicalities of moving critically ill patients to undergo a CT scan are also difficult; thus, a risk‐benefit approach has been taken by some clinicians, reserving this technology for patients with complications of COVID‐19 infection or when other causes of illness, such as pulmonary embolism, are suspected. CT is inferior to ultrasound in showing smaller peripulmonary lesions. On the other hand, ultrasound can produce real‐time, dynamic images and is therefore more advantageous for distinguishing interstitial lesions and showing the distribution of blood flow and angiogenesis in inflammatory lesions.

LUS, when performed with trained clinicians, metanalysis, and review articles, can detect pneumonia with similar accuracy and reliability to chest radiographs.^11^, ^12^ In the ICU setting, LUS was superior to chest X‐ray in detecting pneumonia.^13^ Although CXR has poor sensitivity and specificity compared to chest CT and LUS, it remains the standard protocol for diagnosing the disease. Plain radiographs can miss up to 40% of confirmed COVID‐19 cases due to the nature of the disease, in which lesions are peripherally distributed, and pathology is evident primarily in the terminal alveoli and close to the pleural interface. These areas are well visualized on CT and LUS but are more difficult to see on plain imaging.^14^, ^15^

We used a previously validated scheme of LUS for other diseases, as there is no validated scheme for COVID‐19.^5^ We included scanning of the posterior and lateral zones, where lung lesions are more commonly seen in patients with COVID‐19.^16^ If the patient cannot move from the supine position, the posterolateral part of the chest can usually be scanned by turning the patient to his/her side. As COVID‐19 patients are commonly managed in the prone position, the anterolateral part can also be scanned.

The sonographic appearance of the lungs in patients with COVID‐19 depends on the time course of the illness and other pre‐existing or superimposed conditions. We chose to assess patients with early signs after admission and those with respiratory symptoms because this method is important for early diagnosis, and the results are expected to be of special characteristics to COVID‐19 pneumonia, that could be found in other viral pneumonia, before ARDS or secondary infection development after admission to the ICU. Volpicelli et al and Peng et al. described the signs of COVID‐19 well. Both reported thickening and irregularity of the pleural line, bilateral patchy distribution of multiform clusters of B lines, and multiple small peripheral consolidations.^2^, ^16^ Lesions were located mostly in the lateral fields of both lungs (axillary). The B lines appeared in clusters as separate and coalescent forms, sometimes giving the appearance of a shining white lung. They can arise from one point of the pleural line and from small peripheral consolidations and spread down as rays, maintaining their brightness to the edge of the screen without fading. Compared to B lines caused by cardiogenic pulmonary edema, the B lines observed in noncardiogenic pulmonary edema were more likely to have a nonhomogeneous distribution, spared areas, abnormal pleural lines, reduced or absent lung sliding, and consolidations.^17^ In the case of pulmonary fibrosis secondary to radiation, there was pulmonary edema, making the condition difficult to differentiate from COVID‐19 pneumonia. Otherwise, diffuse irregularities of the pleural line without the typical patchy distribution of B lines are more typical of chronic diffuse interstitial pulmonary diseases, such as fibrosis.

Multiple consolidations of variable size were observed in the subpleural lesions. The subpleural consolidations were bilateral, the echogenicity in the lesions was homogeneous or inhomogeneous, and an air bronchogram sign was visible. The subpleural lesions according to some authors could be due to small pulmonary infarcts.^18^ We had two elderly patients with multiple comorbidities who presented with isolated large lobar consolidation without effusion and with dynamic air bronchograms.

Despite their being negative for COVID‐19, their RT‐PCR results appeared positive. Whether lobar pneumonia was secondary (bacterial) or due to viral infection was difficult to determine with certainty, although lobar pneumonia with dynamic air bronchogram is most often bacterial.^16^

The specificity of the RT‐PCR tests is 100% because the primer design is specific to the genome sequence of SARS‐CoV‐2; however, the incidence of false negatives from nasopharyngeal swab sampling is high.^19^ False negatives result primarily from deficient sampling techniques and inappropriate timing of sample collection in relation to illness onset. We had three cases of false negatives, and LUS was more in keeping with the clinical picture of COVID‐19 infection and other lab tests, such as ferritin level, procalcitonin, d‐dimer, and fibrinogen, and we proceeded to collect a lower respiratory tract sample, which turned out to be positive.

This study's limitation is that it is a single‐center study involving a relatively small number of patients with a sample size that was less than optimal. Although we had a control group of patients with disorders that may be confused with COVID‐19 (non‐COVID‐19 group), we did not include a sufficient number of patients.

## 5 CONCLUSION

Implementation of LUS during the COVID‐19 outbreak is of diagnostic importance. LUS allows the identification of early signs of interstitial pneumonia. LUS patterns that show a combination of the four major signs offer a high degree of sensitivity and specificity compared to RT‐PCR.

## FUNDING

The authors received no financial support for the research, authorship, and/or publication of this article. The research was performed as part of the employment of the authors in the Ministry of Health and Kuwait Oil Company.

## CONFLICT OF INTEREST

The authors declare there is no conflict of interest.

## AUTHOR CONTRIBUTIONS

Conceptualization: zouheir bitar, Ossama Maadarani

Data Curation: Zouheir Bitar, Omar Bamasood

Formal Analysis: Zouheir Bitar, Ossama Maadarani

Funding Acquisition: Huda Alfoudri, Mohammed Shamsah

Investigation: Ossama Maadarani, Omar Bamasood

Methodology: Zouheir Bitar, Ossama Maadarani, Omar Bamasood

Project Administration: Zouheir Bitar, Huda Alfoudri

Resources: Zouheir Bitar, Ossama Maadarani, Omar Bamasood

Software: Zouheir Bitar

Supervision: zouheir bitar,Huda Alfoudri, Mohammed Shamsah

Validation: zouheir bitar, Huda Alfoudri, Mohammed Shamsah

Visualization: Huda Alfoudri, Mohammed Shamsah

Writing‐ Original Draft Preparation: Zouheir Bitar

Writing‐Review & Editing: Zouheir Bitar, Huda Alfoudri, Ossama Maadarani, Omar Bamasood

All authors have read and approved the final version of the manuscript.

Zouheir Bitar had full access to all the data in this study and acts as guarantor for the integrity of the data and the accuracy of the data analysis.

## TRANSPARENCY STATEMENT

The lead author confirms that the manuscript is an honest, accurate, and transparent account of the study being reported; that no important aspects of the study have been omitted; and that any discrepancies from the study as planned have been explained.

## ETHICS APPROVAL AND CONSENT TO PARTICIPATE

This prospective observational study was approved by the Research Ethics Committee of the Ministry of Health in Kuwait (approval number: 2020/1471). Informed consent from the patients was obtained from their legally authorized representatives before enrolment in the study. Images are entirely unidentifiable, and consent for publication is not required.

## Supporting information


**Video S1** B lines arise from one point of the irregular pleural line and from small peripheral consolidations and spread down like rays, maintaining their brightness until reaching the edge of the screen without fading.

**Video S2** Large subpleural consolidation (C) with air bronchogram.

## Data Availability

The datasets used and analyzed during the current study are available from the corresponding author on request.
